# Assessing the Effectiveness of a Far-Red Fluorescent Reporter for Tracking Stem Cells In Vivo

**DOI:** 10.3390/ijms19010019

**Published:** 2017-12-22

**Authors:** Jing Zhou, Jack Sharkey, Rajeev Shukla, Antonius Plagge, Patricia Murray

**Affiliations:** 1 Institute of Translational Medicine, University of Liverpool, Liverpool L69 3BX, UK; J.Zhou18@liverpool.ac.uk (J.Z.); J.Sharkey@liverpool.ac.uk (J.S.); 2 Centre for Preclinical Imaging, University of Liverpool, Liverpool L69 3GE, UK; 3 Alder Hey Children’s NHS Foundation Trust, Liverpool L12 2AP, UK; Rajeev.Shukla@alderhey.nhs.uk

**Keywords:** fluorescent reporter, E2-Crimson, mouse embryonic stem cells, knock-in, in vivo imaging

## Abstract

Far-red fluorescent reporter genes can be used for tracking cells non-invasively in vivo using fluorescence imaging. Here, we investigate the effectiveness of the far-red fluorescent protein, E2-Crimson (E2C), for tracking mouse embryonic cells (mESCs) in vivo following subcutaneous administration into mice. Using a knock-in strategy, we introduced *E2C* into the *Rosa26* locus of an *E14-Bra-GFP* mESC line, and after confirming that the E2C had no obvious effect on the phenotype of the mESCs, we injected them into mice and imaged them over nine days. The results showed that fluorescence intensity was weak, and cells could only be detected when injected at high densities. Furthermore, intensity peaked on day 4 and then started to decrease, despite the fact that tumour volume continued to increase beyond day 4. Histopathological analysis showed that although E2C fluorescence could barely be detected in vivo at day 9, analysis of frozen sections indicated that all mESCs within the tumours continued to express E2C. We hypothesise that the decrease in fluorescence intensity in vivo was probably due to the fact that the mESC tumours became more vascular with time, thus leading to increased absorbance of E2C fluorescence by haemoglobin. We conclude that the E2C reporter has limited use for tracking cells in vivo, at least when introduced as a single copy into the *Rosa26* locus.

## 1. Introduction

During the past two decades, considerable advances have been made in the field of fluorescent imaging technology, especially with regard to fluorescent reporter genes, which are particularly useful because they facilitate long-term cell tracking. In vivo optical imaging enables the study of living organisms by monitoring and characterising physical and biological processes within specific cells or tissues of interest. Fluorescence-based labelling serves as an ideal tool for in vivo imaging in animals. However, due to the photon absorption by tissue haemoglobin (<650 nm wavelength region) and water (>900 nm wavelength region), the majority of visible fluorescence (e.g., green fluorescent protein (GFP) green emitted light) is absorbed within 500 µm of the surface tissue of the recipient animal. To circumvent this, an optical window of approximately 650–900 nm of the near infra-red wavelength region of the spectrum is favourable, which allows maximum depth of fluorescence penetration [1]. In addition, cell nuclei and mitochondria cause light scattering within the optical window [2], whereas the intensity can be reduced by using longer wavelengths [3]. This requires fluorescent probes that have emission spectra within the far-red region (710–850 nm wavelength, which is part of the near-infrared spectrum). E2-Crimson (E2C) is a tetramer derived from DsRed-Express2 with the advantages of fast maturation, high solubility, high photostability and low cytotoxicity. Furthermore, the excitation and emission maxima of E2C are 611 and 646 nm, respectively. This means that it can be excited efficiently by standard far-red lasers routinely used in optical instruments, making it suitable for tracking cells in vivo [4].

Mouse embryonic stem cells (mESCs) have served as invaluable tools for understanding the cellular and molecular mechanisms that regulate mammalian development, and for determining the signalling pathways required for the differentiation of specific cell lineages [5,6,7]. In order to achieve long-term cell tracking, many transgenic mESC lines have been established by using viral transduction of fluorescent reporter genes [8,9,10,11,12,13]. However, the random insertion of reporter genes into chromosomes results in different gene copy numbers and integration sites [11,14,15]. Furthermore, despite the fact that this approach can lead to higher expression levels due to the presence of multiple copies of the transgene, the levels of expression can vary considerably between different cells within the transduced population [9,10,11,12,16,17,18]. There can be a high prevalence of transcriptional silencing of the transgene in vitro and in vivo, which may be due to DNA methylation [19,20,21,22]. There is also evidence that transgenes introduced into mESCs via viral transduction have a tendency to be silenced following differentiation to specific lineages [15].

Gene knock-in has provided a feasible alternative to producing transgenic mESC lines where the plasmid vectors serve as exogenous gene carriers to incorporate the reporter genes into the chromosomal locus of interest, subsequently leading to the disruption of the targeted locus with constant expression of the transgene [23,24]. One of the widely-considered targeting loci is *Rosa26*. Located on mouse chromosome 6, the *Rosa26* locus comprises three exons spanning approximately 9 kb and generates three non-coding transcripts, one of them being a highly conserved anti-sense RNA [25,26]. It was originally identified by Soriano’s group, who using promoter-trap screening, integrated the *β-geo* (fused gene formed from the β-galactosidase (β-gal) and neomycin resistance genes) cassette into mESCs derived from the 129Sv mouse strain via a retroviral targeting vector named ROSAβ-gal (reverse orientation splice acceptor β-gal). The resulting mutants showed constitutive and ubiquitous expression of a single copy of the transgene in all tissues at all pre- and post-natal developmental stages of the germ-line chimeric mice, albeit at different levels of expression. Moreover, none of the progeny exhibited any obvious phenotype [27]. Taken together, these reports highlight the usefulness of *Rosa26* to construct stable reporter mESC lines for tracking cells following transplantation [28].

A common strategy for introducing transgenes into the *Rosa26* locus of mESCs involves a knock-in vector that contains the exogenous reporter gene cDNA sequence preceded by a splice acceptor sequence, a positive selection marker (usually a neomycin resistance gene cassette), the *Rosa26* 5′- and 3′-homology arms (HAs) flanking the aforementioned elements, and a negative selection marker (commonly a diphtheria toxin A subunit, DT-A). During the recombination between the *Rosa26* homology arms, the transgene integrates into the first intron of *Rosa26* at a specific *Xba*I site and is expressed constitutively under the control of the *Rosa26* promoter [25]. Using this strategy, it has been reported that the targeting efficiency of the *Rosa26* locus is around 20% of drug-resistant colonies [26,29], or up to 45% if longer arms of homology were used [30].

*Brachyury* (*Bra*, also known as *T*) is the key marker of the entire primitive streak and is a pan mesodermal marker that is expressed until the stage of tail bud formation, playing an important role in mesoderm development [31,32,33,34,35,36,37,38]. Fehling et al. generated an *E14-Bra-GFP* mESC line to investigate mesodermal differentiation in vitro. In this line, an eGFP mini gene cassette was targeted into the *Bra* locus of E14.1 ESCs, replacing approximately two-thirds of exon 1 of *Bra* [39]. GFP expression was under the control of the *Bra* promoter, which means that cells that undergo mesoderm differentiation are marked by GFP fluorescence [39]. The GFP^+^ cells isolated in vitro have shown the ability to generate mesodermal derivatives including the haematopoietic and cardiac lineages, as well as neuromesodermal progenitors [39,40,41,42].

The aim of this study was to assess the effectiveness of the E2C reporter for in vivo imaging. To this end, we generated a mESC line labelled with combinatorial reporters E2-Crimson and GFP by knocking-in the *E2C* transgene into the *Rosa26* locus of the *E14-Bra-GFP* mESC line based on Soriano’s strategy. After confirming that this genetic modification did not affect the phenotype of the mESCs, we then injected the cells subcutaneously in mice and tracked them using non-invasive fluorescence imaging.

## 2. Results

### 2.1. Rosa26 Knock-in of E2-Crimson Transgene and Analysis of E2C Expression in Screened Positive Clones

**Figure 1:**
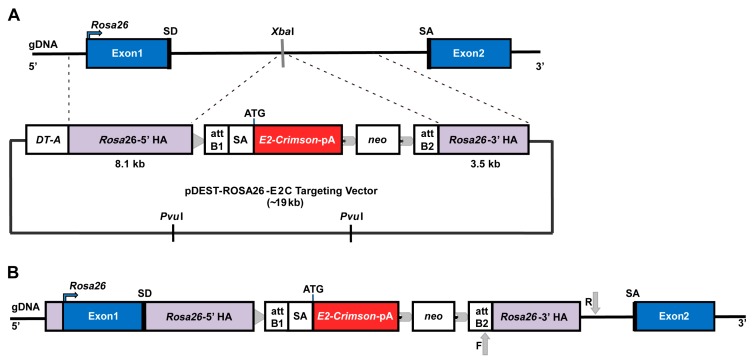
Schematic illustration of the *Rosa26* knock-in strategy. (**A**) *E14-Bra-GFP* mESC *Rosa26* chromosome structure and pDest-ROSA26-E2C targeting vector. The vector contains two homology arms (HAs) that flank the *E2-Crimson* cassette, which has a polyadenylation (pA) signal, and a downstream positive drug selection marker *neo* (neomycin resistance). The insertion site in between the HAs is identified by the *Xba*I restriction enzyme site in the *Rosa26* locus. A lethal negative selection marker DT-A is placed in the 5′ upstream region adjacent to the targeting arm. The *Pvu*I restriction enzyme sites are located in the vector backbone. When linearised for gene targeting, most of the vector backbone is removed following *Pvu*I restriction digestion; (**B**) *E14-Bra-GFP* mESC *Rosa26* locus with correctly targeted insertion of linearised pDest-ROSA26-E2C targeting vector. In the correct recombinants, *E2C* cDNA is introduced into the *Rosa26* locus and is expressed under the control of the *Rosa26* promoter. A loxP site (indicated with a grey triangle) is located next to the 5′ HA. Flippase recombinase target (FRT) sites (indicated with grey arrow heads) flanking the *neo* cassette are designed for optional removal of the *neo* cassette. Grey arrows show the forward and reverse primer-binding sites for the PCR screen of correct homologous 3′-HA recombination.

**Figure A1:**
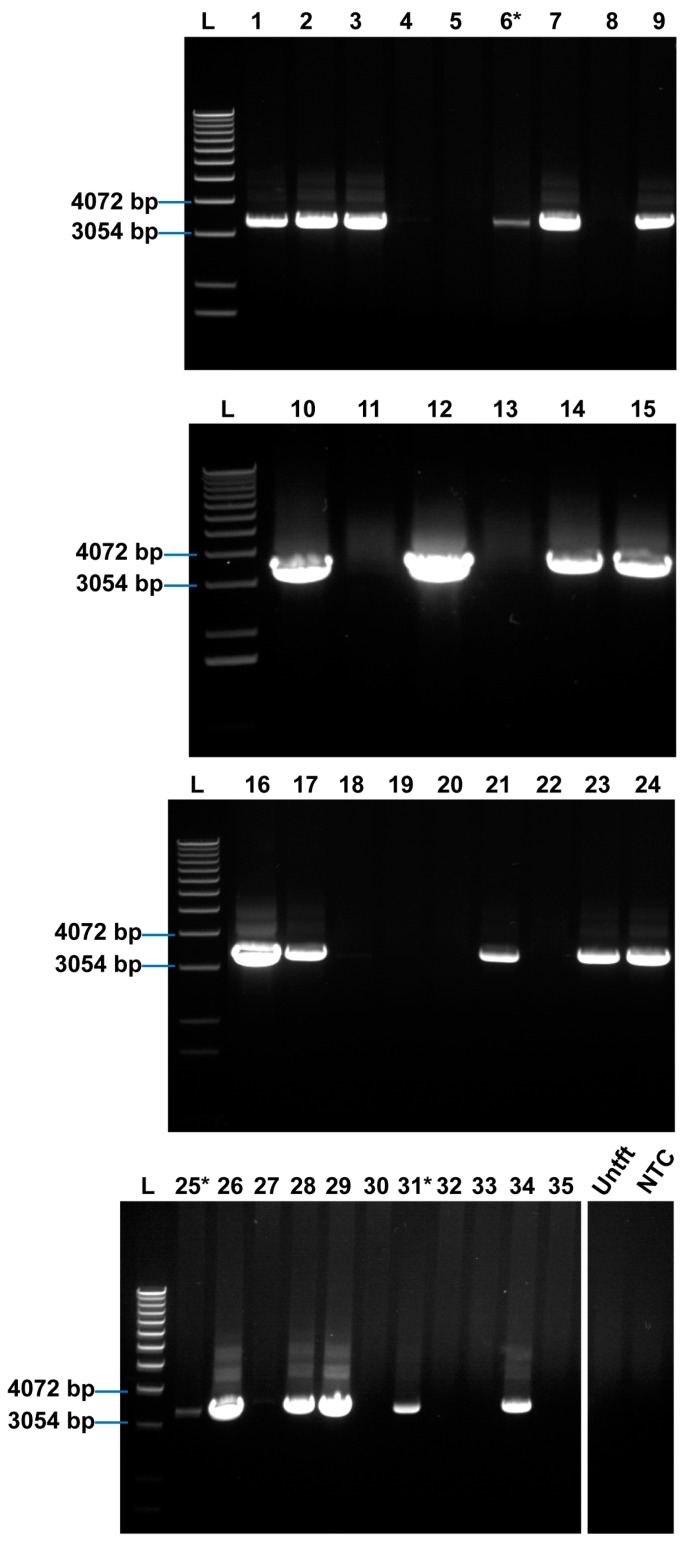
0.8% agarose gel electrophoresis of a total of 35 viable clones screened by 3′-HA PCR. 500 ng gDNA templates (numbered as 1 to 35) were amplified at the annealing temperature of 65 °C for 30 cycles, with gDNA template from untransfected (Untft) *E14-Bra-GFP* mESCs cultured on gelatine as a negative control. A non-template control (NTC) was also included where template gDNA was substituted by nuclease-free water. Eighteen out of 35 viable clones show bright positive bands and three show weaker bands (indicated by asterisks) of the correct size of 3380 bp. L, 1 kb ladder.

The *Rosa26* knock-in strategy is shown in Figure 1. Following electroporation of *E14-Bra-GFP* mESCs with the targeting plasmid and G418 selection, 35 colonies were expanded and analysed by long-range PCR for correct insertion at the *Rosa26* locus. Twenty-one clones showed the correct PCR product size of approximately 3.4 kb and thus were considered positive recombinants. Positive recombinants comprised just over 50% of the resultant clones (Figure A1).

**Figure 2:**
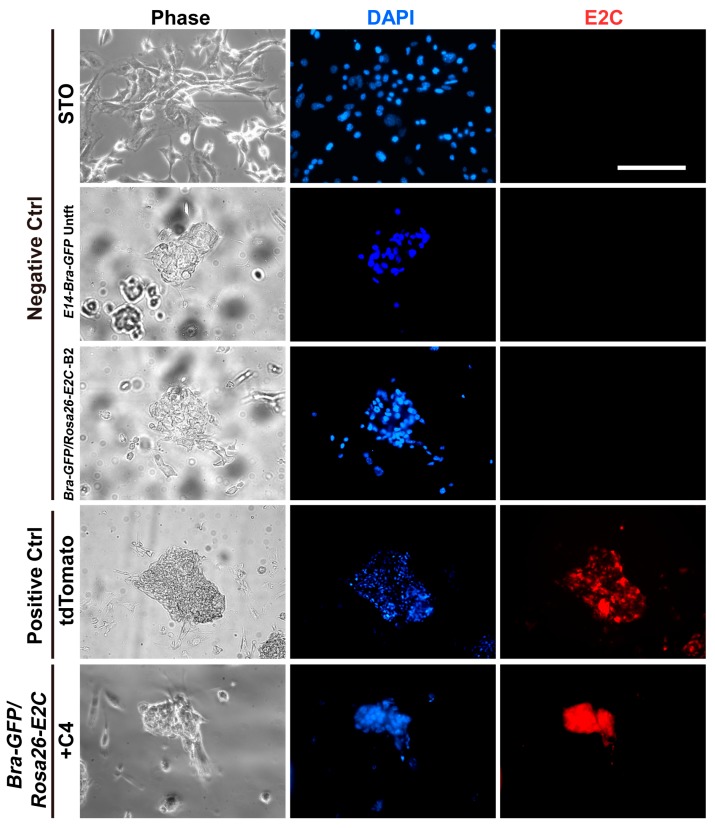
E2C expression was examined by fluorescence microscopy in 3′-HA PCR-screened positive and negative clones. Fluorescence signal was detected within the positive clone named +C4. tdTomato-transduced *E14-Bra-GFP* mESCs were used as a positive control with STO feeder cells, untransfected (Untft) *E14-Bra-GFP* mESCs and a negative clone named −B2 comprised the negative controls. All samples were counter-stained with the nuclear stain DAPI. Scale bar for all graphs, 100 µm.

**Figure 3:**
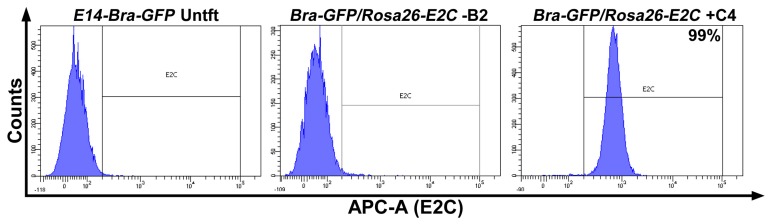
Flow cytometry analysis of E2C expression of the selected 3′-HA PCR-screened positive clone named +C4. Untransfected (Untft) *E14-Bra-GFP* mESCs and a negative clone named −B2 was used as the negative controls. 10,000 events were counted for each sample.

The 18 positive and three randomly selected negative clones were individually expanded from the 96-well cryopreserved plates. Viable clones were expanded and investigated for expression of E2C via fluorescence microscopy. Five positive clones were found to be fluorescent, and their intensity was comparable to that of tdTomato-transduced *E14-Bra-GFP* mESCs (produced in-house). No fluorescence signal was detected among the negative clones (Figure 2). To test whether the positive clones constituted homogeneous E2C-expressing cell populations, four of the E2C-expressing clones, namely +B5, +C4, +D1 and +E3 were selected for flow cytometry. The average E2C signal intensity of all 4 clones was approximately 10^3^. For clone +C4, 99% of the population expressed E2C, indicating very high purity (Figure 3). +C4 was therefore selected for subsequent experiments.

### 2.2. Stemness and Differentiation Potential of Bra-GFP/Rosa26-E2C mESC Reporter Line

**Figure 4:**
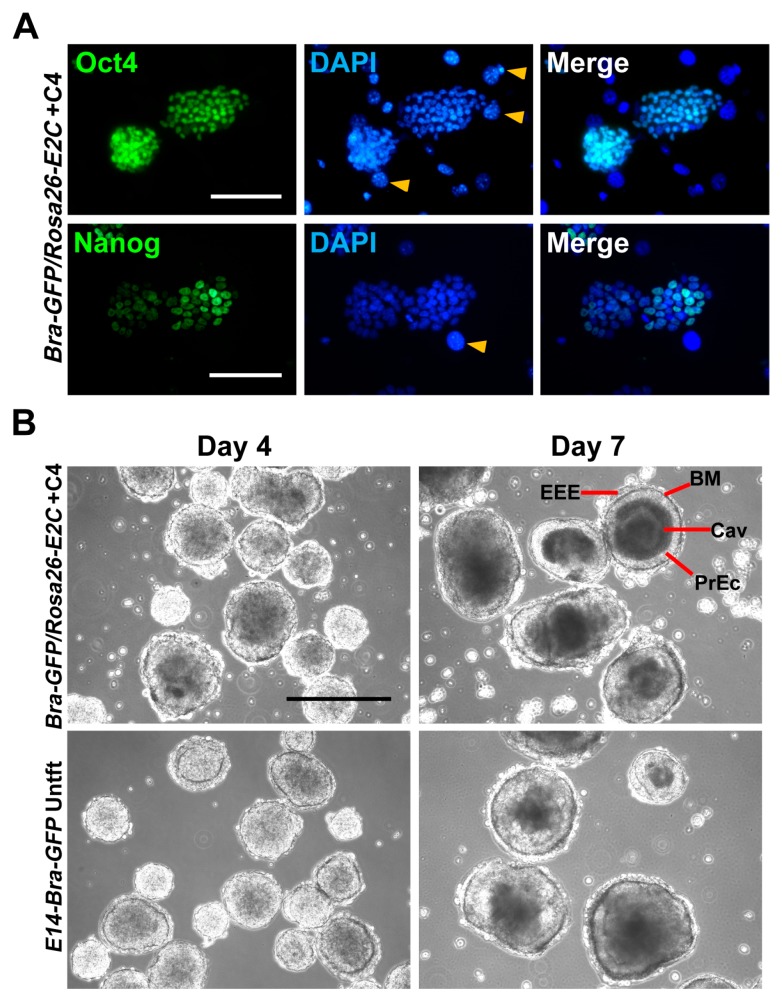
(**A**) Expression of stemness markers Oct4 and Nanog was confirmed by immunofluorescent staining of *Bra-GFP/Rosa26-E2C* mESC clone +C4. All samples were counter-stained with DAPI. Yellow arrow heads show STO feeder cell nuclei; (**B**) Differentiation potential of clone +C4 was confirmed by typical EB formation. Cells were seeded in suspension culture dishes up to day 7. PrEc, primitive ectoderm; EEE, extraembryonic endoderm; BM, basement membrane; Cav, cavity. Scale bars, 100 µm (**A**); 400 µm (**B**).

To ensure the knock-in of *E2C* did not affect self-renewal or differentiation capacity, the cells were assessed for the expression of stemness markers and their ability to generate embryoid bodies (EBs). To examine the expression of stemness markers, the clone +C4 was immunostained for Oct4 and Nanog. The results showed strong nuclear expression of these two pluripotent stem cell markers, suggesting that the knock-in of *E2C* had not affected mESC phenotype (Figure 4A).

To evaluate whether typical EBs can be formed following the *E2C* knock-in manipulation, +C4 mESCs were plated in bacterial petri dishes and cultivated for seven days in EB medium. Aggregates were observed during the first 24 to 48 h and cavitation was observed in the majority of EBs by day 7 (Figure 4B). Basement membranes, primitive ectoderm and extraembryonic endoderm were also identifiable by day 7 in the cavitated EBs, suggesting that the +C4 cells had retained their ability to differentiate to different lineages (Figure 4B).

### 2.3. Quantitative Analysis of E2C Fluorescence Signal In Vivo

**Figure 5:**
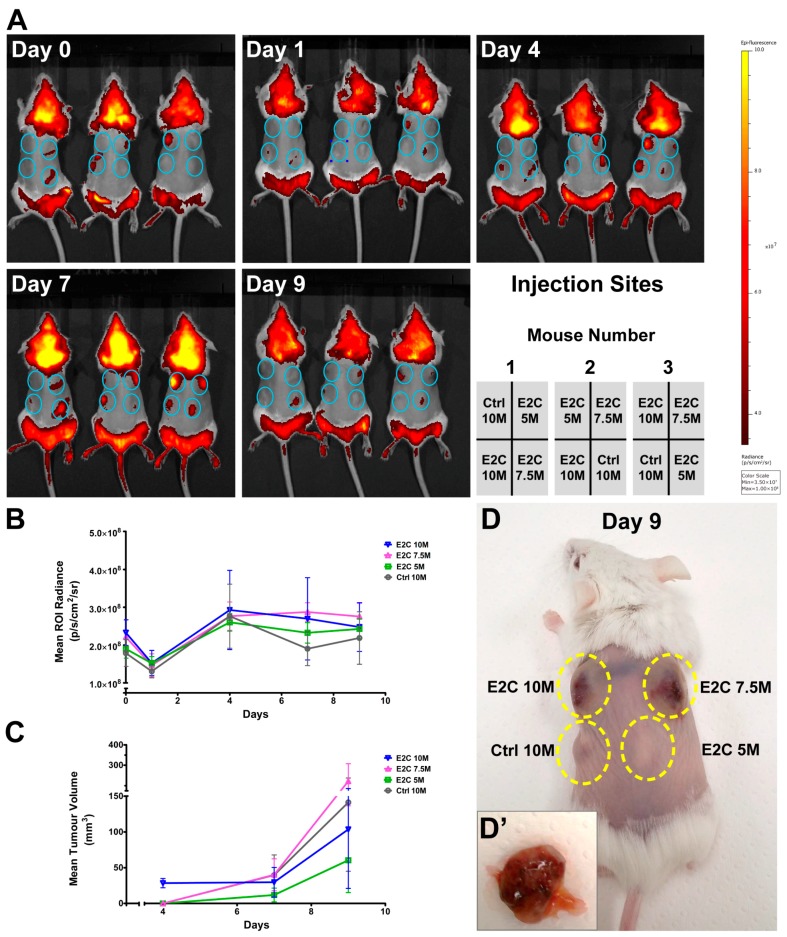
(**A**) IVIS images in vivo at days 0, 1, 4, 7 and 9 post injection of E2C^+^ cells into the dorsal flanks of severe combined immunodeficient (SCID) mice. The emission filter of 605–660 nm wavelengths was applied to detect the fluorescent signal generated from *Bra-GFP/Rosa26-E2C* mESCs. The untransfected *E14-Bra-GFP* cells were used as negative controls. Blue circles show the injection sites of cells in the mice. Data are displayed in radiance units (p/s/cm^2^/sr); (**B**) Growth curves obtained based on the changes of mean region of interest (ROI) radiance post injection. ROI radiance was generated by IVIS software for the time points of day 0, 1, 4, 7 and 9; (**C**) Growth curves obtained based on the changes of tumour volume post injection. Tumour volume was calculated based on the manual measurements of tumours on day 4, 7 and 9; (**D**) Representative presence of tumours (dashed lines) from *Bra-GFP/Rosa26-E2C* mESCs in SCID mice (number 3) that formed from 5 × 10^6^, 7.5 × 10^6^ and 10 × 10^6^ injected cells on day 9 post injection. The untransfected *E14-Bra-GFP* cells were used as a negative control (10 × 10^6^ cells); (**D′**) Dissected day-9 tumour that formed from 7.5 × 10^6^ injected *Bra-GFP/Rosa26-E2C* mESCs showed high degree of vascularisation. M represents million (×10^6^). Error bars represent ±SD (**B**) and ±SEM (**C**), respectively (*n* = 3).

*Bra-GFP/Rosa26-E2C* mESCs suspended in phosphate-buffered saline (PBS) were injected subcutaneously in randomly selected sites in each mouse in an injection volume of 100 µL (number of injected cells: 5 × 10^6^, 7.5 × 10^6^ and 10 × 10^6^). Controls comprised 10 × 10^6^ untransfected *E14-Bra-GFP* mESCs (Figure 5A).

E2C fluorescence emitted from the reporter cell line in some injection sites was noticeable in vivo immediately following injection. In all experimental groups that were administered with *Bra-GFP/Rosa26-E2C* mESCs, the E2C decreased by 24 h post injection. Detectable fluorescence of E2C increased afterwards from this time-point and peaked on day 4 (Figure 5B). Surprisingly, although E2C signal intensity decreased between days 4 and 9 (Figure 5B), visual inspection suggested that the tumours were increasing in size (Figure 5C), with palpable tumours being present by day 4 (Figure 5D). By day 7, several tumours developed reddish-blue discolouration, which was probably due to the high degree of vascularisation (Figure 5D′). Despite this, there was no significant difference in the fluorescence intensity in tumours generated by the reporter cells and untransfected cells (negative controls) within the nine-day growth period (Figure 5B).

Quantitative analysis in terms of tumour size and mean region of interest (ROI) radiance are shown in Tables S1 and S2, respectively. To determine if the emitted fluorescence intensity correlated with tumour size, tumour volume was measured on days 4, 7 and 9. Tumour growth curves were then constructed. The measurement data here displayed a distinct increase in tumour size during day 4 and 9. The increase in tumour volume for the 7.5 × 10^6^ dose of E2C reporter mESCs demonstrated an approximately 4-fold change compared to that of the 5 × 10^6^ cell dose. However, a noticeable increase in tumour volume was observed in all cases, irrespective of the administration dose (Table S2).

### 2.4. Histopathology and Immunofluorescence Analyses of Tumours

**Figure 6:**
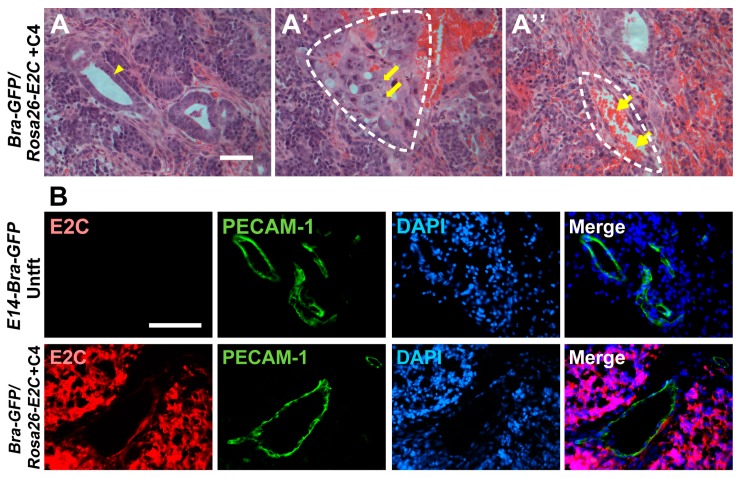
Histopathology and immunofluorescence analyses of tumours derived from the mESCs. (**A**–**A′′**) Haematoxylin and eosin (H&E) staining was performed on paraffin sections prepared from the day-9 tumours. Arrow heads show epithelial structure (**A**), thick arrows show chondrocyte-like cells (**A′**) and thin arrows show red blood cells (**A′′**). Dashed lines show the structures of chondrogenic-like (mesoderm-like) differentiation (**A′**) and blood vessels (**A′′**); (**B**) Immunostaining for E2C (red) and PECAM-1 (green) in tumours derived from *Bra-GFP/Rosa26-E2C* mESCs or untransfected controls (Untft). Dual immunostaining of frozen sections prepared from tumours harvested at day 9 showed that all cells, except the vasculature and blood cells, within the tumours were derived from the E2C^+^ mESC, which stained positively for E2C. On the other hand, the endothelial cells within the tumours did not stain positively for E2C, indicating they were derived from the host animals. Tumours developed from the untransfected *E14-Bra-GFP* mESCs were used as controls. Scale bars, 50 µm (**A**–**A′′**); 100 µm (**B**).

At the study end point, the mESC tumours were harvested and paraffin sections were analysed by a pathologist to confirm whether the tumours resembled teratomas. Histologically, tumours were composed of lobules and nests of primitive cells, focally forming tubular structures with central lumina and rosette-like structures with some polarisation of lining cells but no lumen. The tubular formations resembled primitive ectodermal epithelia, and rosette-like structures were reminiscent of neuroepithelium. In addition, foci of immature mesenchyme and pale islands suggestive of early chondroid differentiation were evident. There were no well-differentiated elements, nor any morphologically identifiable yolk sac or trophoblastic differentiation. In a given context it is consistent with very immature teratoma, featuring ectodermal elements including immature neuroepithelium and mesodermal elements featuring immature mesenchyme with chondroid islands (Figure 6A,A′). The tumours also appeared to be well vascularised and contained abundant erythrocytes (Figure 6A′′).

To determine if all of the differentiated cells within the tumours continued to express E2C, and whether the tumour vasculature was derived from the mESCs or the host, immunofluorescence staining for E2C and the endothelial marker, PECAM-1, was undertaken. The results showed that most of the tumour cells, including the epithelial and chondrocyte-like cells, expressed E2C, indicating that the transgene expression was stable over this time-frame. As expected, there was no evidence of E2C expression in the control tumours (Figure 6B). Platelet endothelial cell adhesion molecule (PECAM-1) staining showed that none of the endothelial cells within the tumour stained positively for E2C, indicating that they were all derived from the host (Figure 6B).

## 3. Discussion

In this study, an E2C-expressing *E14-Bra-GFP* mESC reporter line (clone +C4) was generated by knocking-in *E2C* into the *Rosa26* locus. Using the *Rosa26* targeting vector generated by Aizawa’s group [30], which contains long 5′- and 3′-homologous arms and a DT-A counter-selection cassette against random integration, we achieved a high targeting frequency of 51%. Although targeted integration at the *Rosa26* locus generally introduces only a single copy of the transgene into the *Rosa26* allele [27], leading to lower expression levels than what can be achieved with lentiviral transduction, there is a lower tendency for transgenes within the *Rosa26* locus to become silenced following differentiation of the mESCs [15]. Therefore, this approach could be more suitable for tracking cells over the long-term.

To assess the feasibility of imaging the E2C mESC reporter cells in vivo using fluorescence imaging, we injected three cell doses subcutaneously into SCID mice and imaged over a nine-day time course. It was found that, even when the animals were imaged immediately following cell administration, it was not always possible to detect the E2C signal. Thus, the main conclusion from this experiment is that the E2C is not an effective in vivo reporter in this context (i.e., when introduced as a single copy into the *Rosa26* locus), and imaging the E2C reporters in the internal organs would not be feasible due to signal attenuation with increasing depth. In contrast to our findings, Christensen and colleagues were able to detect E2C emitted from tumour cells injected into the rat lung [43]. The likely explanation is that they used lentiviral technology to generate the reporter cells, which would have introduced multiple copies of the *E2C* transgene into each cell. Furthermore, the E2C was expressed under the strong constitutive cytomegalovirus (CMV) promoter. However, this promoter is not suitable for constitutive expression in mESCs as it tends to be downregulated during propagation and differentiation [44].

In cases where the cells could be detected at day 0, it was found that E2C signal intensity decreased on day 1 before increasing between days 4 and 7. The reduction in signal at day 1 is probably due to cell death. SCID mice are deficient in generating T and B lymphocytes and thus are unable to launch an adaptive immune response. However, they still have an intact innate immune system, comprising neutrophils, macrophages and natural killer cells that might contribute to the death of injected mESCs [45]. Another possible reason is that many of the mESCs may simply die due to the sudden change in their micro-environment, as it is well-known that these cells require specific culture conditions for their propagation. The increase in fluorescence observed in most tumours from days 4 to 7 probably reflected the fact that, over this time course, there was an increase in tumour volume. Interestingly, although the tumour volume continued to increase from days 4 to 7, in most cases, there was a decrease in fluorescence. This was most likely due to the fact that the tumours became highly vascularised. H&E staining of tumour sections showed a high number of red blood cells within the blood vessels. Since haemoglobin plays a major role in photon absorption within the <650 nm wavelength region, it is likely that E2C fluorescence was partially absorbed by the red blood cells in the tumours [1].

None of the mice injected with control untransfected mESCs were expected to display any E2C fluorescence signal. Of note, some background signal was observed in the control tumour of mouse number 3 on days 4 and 7 post injection. This artefactual signal is likely due to photon scattering and autofluorescence from the skin abutting the tumour [46].

H&E histopathological examination of the tumours showed that they resembled immature teratomas, which typically have abundant neuroepithelial cells [47] as well as derivatives of other germ layers, such as chondrocytes [48]. The abundant epithelial structures observed in the tumours are likely to resemble either primitive ectoderm epithelia or neuroepithelia. In the developing embryo, some of the primitive ectoderm cells that do not egress through the primitive streak directly give rise to neuroepithelial cells [49]. Morphologically, the primitive ectoderm and early neuroepithelial cells closely resemble each other. Chondrocyte-like cells were also present in the tumours, which would be consistent with them being immature teratomas. Endothelial cells were also identified lining blood vessels, but dual immunostaining for E2C and PECAM-1showed that these cells were derived from the host.

There are numerous reports showing that following subcutaneous implantation or implantation under the kidney capsule, mESCs will typically form mature teratomas that contain various types of well-differentiated cells derived from the three germ layers, and can even generate endothelial cells that form part of the vasculature of the teratoma [50]. However, in these studies the teratomas were allowed to develop over a four-week period instead of just nine days. Therefore, the fact that teratomas in this study were immature is probably due to the shorter time that they were allowed to develop in vivo.

To summarise, the results obtained from imaging the reporter cells after subcutaneous implantation indicate that the emitted E2C fluorescence is weak and it would not be feasible to image the cells in internal organs such as the kidney. Therefore the focus of future applications of this reporter cell line could be within the field of in vitro imaging and tracking.

## 4. Materials and Methods

### 4.1. Sub-Culture of STO and mESCs

STO feeder cells (ATCC, SCRC-1049) were maintained in 10-cm tissue culture dishes in culture medium consisting of Dulbecco’s Modified Eagle’s Medium (DMEM) (D6546, Sigma-Aldrich, St. Louis, MO, USA), 100 mL/L foetal bovine serum (FBS) (Gibco, 10270, Thermo Fisher Scientific, Waltham, MA, USA), 10 mL/L MEM non-essential amino acid (Sigma-Aldrich, M7145) and 10 mL/L l-glutamine (Sigma-Aldrich, G7513) at 37 °C in a humidified incubator with 5% CO_2_. Cells were passaged twice per week at a split ratio of 1:4–1:6 until reaching passage 15. *E14-Bra-GFP* mESC line used in this study was a generous gift from Georges Lacaud at the Paterson Institute for Cancer Research, Manchester. tdTomato-transduced *E14-Bra-GFP* mESCs were from our in-house stock. mESCs were maintained in the 6-well feeder plates in culture medium consisting of DMEM, 150 mL/L FBS (Sigma-Aldrich, F2442), 10 mL/L MEM non-essential amino acid, 10 mL/L l-glutamine, 0.1 mmol/L β-mercaptoethanol (Gibco, 31350) and 1000 U/mL mouse leukaemia inhibitory factor (mLIF) (ESG1107, Merck Millipore, Billerica, MA, USA) at 37 °C in a humidified incubator with 5% CO_2_. Cells were passaged every other day at a split ratio of 1:6–1:10 until reaching passage 40.

### 4.2. Preparation of Mitomycin-C-Inactivated STO Feeder Cells

24 h prior to the inactivation, the STO culture medium was replaced with fresh medium. For the inactivation, mitomycin-C (Sigma-Aldrich, M4287) was added to give a final concentration of 20 µg/mL. The dishes were incubated for 2 h and rinsed with 1× PBS (without Ca^2+^ and Mg^2+^). Cells were collected and plated into gelatinised tissue culture dishes at a density of 5 × 10^4^ cells/cm^2^. Prior to plating the mESCs, feeder cells were rinsed once with PBS.

### 4.3. Preparation and Linearisation of Knock-in Construct

The targeting construct pDEST-ROSA26-E2C was generated by using the pROSA26-STOP-Dest vector, which was kindly provided by Shinichi Aizawa’s group (RIKEN, Tokyo, Japan) [30]. The pROSA26-STOP-Dest plasmid was derived from pROSA26-Dest and contains homologous sequences of 8.1 and 3.5 kb, respectively, for reporter gene knock-in into the *Rosa26* intronic *Xba*I site [25,29]. In addition, it contains a PGK-DTA-pA cassette located outside of the 5′-homologous sequence for diphtheria toxin expression and counter-selection of mESCs. Briefly, a splice acceptor cassette, the E2-Crimson open reading frame and an FRT-flanked neomycin phosphotransferase expression cassette were assembled in the Gateway plasmid pENTR2B (Thermo Fisher Scientific, A10463). Using the Gateway Clonase system (Thermo Fisher Scientific), this cassette was then transferred into pROSA26-STOP-Dest as previously described [29]. Finally, the loxP flanked STOP cassette of the plasmid was removed by transformation into *E. coli* strain 294-Cre (A111, Gene Bridges, Heidelberg, Germany), which resulted in a single loxP site remaining 5′of the attB1 site and E2-Crimson cassette. To linearize the targeting construct, 50 µg pDEST-ROSA26-E2C plasmid DNA was digested by *Pvu*I (Thermo Fisher Scientific, FD0624). The targeting DNA fragment was separated by gel electrophoresis and extracted using Wizard^®^ SV Gel and PCR Clean-Up System (A9282, Promega, Madison, WI, USA). DNA concentrations and purity were determined using a NanoDrop 2000 spectrophotometer (Thermo Fisher Scientific, ND-2000). Where necessary, an ethanol precipitation was performed to further concentrate linearised DNA. Purified linearised DNA was dissolved in 1× TE buffer.

### 4.4. Generation of the Knock-in mESC Reporter Line

The *E14-Bra-GFP* mESCs at passage *7* were expanded in 10 cm feeder dishes in mESC culture medium. When reaching approximately 70% confluence, cells were trypsinised, pelleted and resuspended in 1× PBS (without Ca^2+^ and Mg^2+^). The cell suspension was mixed with linearised targeting construct (DNA final concentration 1 µg/µL) and was transferred into cuvettes (800 µL each) for electroporation (Bio-Rad Gene Pulser with capacitance extender, 240 V, 500 µF, Bio-Rad Laboratories, Hercules, CA, USA). Cell suspension was transferred to 10 cm feeder dishes and incubated at 37 °C in a humidified incubator with 5% CO_2_. Two days post electroporation, G418 (Sigma-Aldrich, A1720) was added into the culture medium at a final concentration of 200 µg/mL, initiating the counter-selection process. Medium was changed daily and supplemented with G418 until day 9 of the selection. Viable clones were picked individually and transferred to 96-well non-treated round-bottom plates containing 1× trypsin/EDTA. Colonies were dissociated and mESC medium was added to neutralise the trypsinisation. The solution was then transferred to 96-well feeder plates and incubated at 37 °C in a humidified incubator with 5% CO_2_. After 24 h expansion, cells in each well were trypsinised and neutralised with mESC medium to reach a total volume of 200 µL per well, half was transferred to gelatinised 24-well tissue culture plates for screening purpose, whilst half was sealed and frozen at −80 °C for subsequent expansion.

### 4.5. Genomic DNA Extraction of mESCs

mESC samples of viable clones were collected in 1.5 mL microfuge tubes and resuspended in lysis buffer containing 100 µg/mL Proteinase K (Sigma-Aldrich, P6556) for an incubation at 55 °C overnight. One volume of 2-propanol was added followed by centrifugation at 16,000× *g* for 1 min to obtain the genomic DNA (gDNA) pellets. gDNA pellets were washed twice with 70% ethanol and air dried. They were then re-dissolved in 1× TE buffer at 4 °C overnight followed by 65 °C for 15 min. DNA concentrations and purity was measured using the NanoDrop 2000 spectrophotometer (Thermo Fisher Scientific).

### 4.6. 3′-Homology Arm PCR Analysis

3′- homology arm (HA) PCR analyses were performed on the gDNAs of viable colonies using a GoTaq^®^ Long PCR reaction system (Promega, M4021) according to the manufacturer’s manual. gDNA template from untransfected *E14-Bra-GFP* mESCs served as a negative control. A non-template control was also included where template gDNA was substituted by nuclease-free water. PCR primer sequences were: 5′-GGCTTCTGAGGCGGAAAGA-3′ (forward); 5′-CAACAATCAGCCTAAGGTAG-3′ (reverse). The PCR programme was set up as follows: hot start at 95 °C for 2 min, followed by PCR steps comprising denaturation at 94 °C for 30 s, annealing at 65 °C for 30 s, elongation at 65 °C for 4 min, and final extension at 72 °C for 10 min, for 30 cycles. Products were examined on a 0.8% agarose gel.

### 4.7. Analysis of E2C Expression in the PCR Screened Positive Clones with Fluorescence Microscopy

Screened clones were plated on gelatin for 48 h and then re-plated on gelatinised eight-chamber slides. When reaching required colony size, they were fixed with 4% paraformaldehyde (PFA) followed by counter-staining with 4′,6-diamidino-2-phenylindole (DAPI) (Thermo Fisher Scientific, D1306, 1/100,000) as for immunofluorescence staining. Slides were examined by the Leica DM2500 (Leica, Wetzlar, Germany) fluorescence microscope with the 561 nm laser and data were acquired by the Leica Application Suite (LAS, Leica) integrated software. STO feeder cells as well as untransfected and tdTomato-transduced *E14-Bra-GFP* mESCs were included as negative and positive controls, respectively.

### 4.8. Flow Cytometry Analysis

Single cell suspension in 1× PBS (1 × 10^6^ cells/mL) was acquired from *Bra-GFP/Rosa26-E2C* cells. Prior to the analysis, the suspension was filtered using a 40-µm strainer (BD Falcon, 352340, Hunter Scientific, Saffron Walden, UK). The percentage of E2C^+^ cells were analysed using a BD FACSCanto (BD Biosciences, San Jose, CA, USA) flow cytometer. Untransfected *E14-Bra-GFP* mESCs were used as a negative control. Data output was performed using BD FACSDiva (version 6.1.3) software.

### 4.9. Embryoid Body Formation

To form EBs, early passages of mESCs maintained in STO feeder plates were sub-cultured in feeder-free gelatinised tissue culture plates for 48 h. Cells were collected and resuspended in mouse EB medium consisting of DMEM, 100 mL/L FBS (Sigma-Aldrich, F2442), 10 mL/L MEM non-essential amino acid, 10 mL/L l-glutamine and 0.1 mmol/L β-mercaptoethanol. Cells were then plated in 90-mm bacterial petri dishes (Sterilin, 101VR20, Thermo Fisher Scientific) at the seeding density of 1.25 × 10^5^ cells/mL. The EBs were maintained for up to 9 days. Medium was changed every other day. Each EB dish was split 1:2 on day 3 post plating. EB morphology was examined at days 4 and 7. Experiments were performed in three independent biological replicates.

### 4.10. Administration and Imaging of Bra-GFP/Rosa26-E2C

*Bra-GFP/Rosa26-E2C* and untransfected *E14-Bra-GFP* mESCs were harvested from the feeder plates and expanded in 10 cm gelatinised tissue culture dishes and cultured for 48 h to reach the required density. The cell resuspensions in 1× PBS were stored on ice immediately prior to in vivo administration. Three female CB17 severe combined immunodeficient (SCID) mice (Charles River, Wilmington, MA, USA) were housed in accordance with the guidelines. Experiments were performed following the approved guidelines under a UK Home Office licence (Licence Number: 70/8741; Approval Date: 06/10/2015) under the Animals (Scientific Procedures) Act 1986 and approved by the University of Liverpool Animal Ethics Committee. At the age of 8–10 weeks old, mice were used for mESCs injection. Animals were anaesthetised with isoflurane, shaved and depilated to remove fur from the torso. mESCs in 100 µL of 1× PBS were administered to the dorsal flank of individual mouse via subcutaneous injection at 4 different positions (top left, top right, bottom left and bottom right of the torso). The following doses of 10, 7.5 and 5 × 10^6^/100 µL *Bra-GFP/Rosa26-E2C* mESCs were injected into three of the positions in a random manner. 10 × 10^6^/100 µL untransfected *E14-Bra-GFP* mESCs were individually injected alongside the *Bra-GFP*/*Rosa26-E2C* engrafts in the same mouse as negative controls. The implantation pattern was in keeping with the suggested UK NC3Rs (National Centre for the Replacement, Refinement, and Reduction of Animals in Research) experimental design, which recommends randomisation to ensure that observed effects on the growth of the implanted cells is not site-dependent. Experiments were performed in three independent biological replicates. Data of the signal intensity in terms of the radiance measured at the regions of interest (ROI) were acquired on days 0, 1, 4, 7 and 9 post injection using the IVIS^®^ Spectrum In Vivo Imaging System (PerkinElmer, Waltham, MA, USA) and analysed with its Living Image^®^ software (version 4.5).

### 4.11. Tumour Volume Measurement

Following the injection, the tumour size was measured using a digital calliper on day 4, 7 and 9 in terms of the length, width and height of the tumours. The length was measured in the direction of the dorsal longitude; the width was measured along the dorsal latitude; and the height was measured perpendicularly in between the surface of the back and the upper surface of the tumours. A mathematical model of solid tumour volume calculation has previously been constructed based on the assumption that tumours are hemi-ellipsoid in 3D shape. Therefore, the volume is calculated from the measurements of tumour length, width and height using the following equation [51,52]:

(1) V=πlwh/6

where *V* is the tumour volume, and *l*, *w*, *h* are the length, width, and height of the tumour, respectively.

### 4.12. Tumour Fixation

Mice were sacrificed on day 9, when the tumours reached the size limit according to the Home Office guidelines. Tumours were harvested immediately afterwards and fixed with 4% PFA at 4 °C overnight. If necessary, larger tumours were vertically cut into small pieces prior to fixation.

### 4.13. Tumour Histopathological Analysis

Fixed tumours were rinsed with 1× PBS and transferred to 70% ethanol followed by a general paraffin embedding process [53]. Then 5-µm sections were stained with haematoxylin and eosin (H&E) followed by histopathological examination.

### 4.14. Immunofluorescence Staining of Stemness Markers

*Bra-GFP*/*Rosa26-E2C* and untransfected *E14-Bra-GFP* mESC lines were sub-cultured from STO feeder layers to gelatinised tissue culture dishes for 48 h. They were then plated into gelatinised 8-chamber slides or 35 mm dishes and fixed with 4% PFA when reaching the required density. Samples were blocked in 10% serum solution containing 0.1% Triton-X 100 at room temperature for 1 h and then incubated with the primary antibody solutions (Oct4: Santa Cruz, CA, USA, sc-5279, 1/500; Nanog: Abcam, Cambridge, UK, ab80892, 1/500) at 4 °C overnight. They were rinsed with 1× PBS and incubated with Alexa Fluor^®^-labelled secondary antibody solution (Thermo Fisher Scientific, 1/1000) at room temperature for 2 h. All samples were then counter-stained with DAPI at room temperature for 15 min. Slides were mounted with DAKO fluorescent mounting medium (S3023, Agilent Technologies, Santa Clara, CA, USA) and sealed by nail polish. Controls were also included to check for non-specific binding of secondary antibodies, and these comprised samples where primary antibodies were omitted. Data were acquired using a Leica DM2500 or DMIRB fluorescence microscope and the Leica Application Suite (LAS, Leica) integrated software.

### 4.15. Immunofluorescence Staining of Frozen Tumour Sections

Fixed tumours were rinsed with 1× PBS followed by a concessive immersing process of 15% and 30% sucrose at 4 °C overnight, respectively. Tumours were then embedded with embedding resin and frozen in cryostat section machine. Then 10-µm sections were blocked in 10% serum solution(s) at room temperature for 1 h followed by incubation with E2-Crimson (Clonetech, 632496, 1/1000, Takara Bio USA, Inc., Mountain View, CA, USA) and PECAM-1 (BD Pharmingen, 550274, 1/1000, Becton, Dickinson and Company, Oxford, UK) primary antibodies at 4 °C overnight. They were rinsed with 1× PBS and then incubated with Alexa Fluor^®^-labelled secondary antibody solutions (Thermo Fisher Scientific, 1/1000) at room temperature for 2 h. The remaining protocols followed those for stemness markers staining in 4.14. Data were acquired using a Leica DM2500 fluorescence microscope and the Leica Application Suite (LAS, Leica) integrated software.

### 4.16. Statistical Analysis

Data were analysed using GraphPad Prism (GraphPad Software, La Jolla, CA, USA, version 5.01) and processed data were plotted by GraphPad Prism. For the statistical analysis of all the data, the same number/replicates were analysed. Basic functions used were: mean, standard deviation (SD), standard error of the mean (SEM) and Student’s *t*-test.

## Supplementary Materials

Supplementary materials can be found at www.mdpi.com/1422-0067/19/1/19/s1.

## Abbreviations

DAPI	4′,6-diamidino-2-phenylindole
DT-A	Diphtheria toxin A
E2C	E2-crimson
EB	Embryoid body
FBS	Foetal bovine serum
HA	Homology arm
H&E	Haematoxylin and eosin
mESC	Mouse embryonic stem cell
PBS	Phosphate-buffered saline
PECAM-1	Platelet endothelial cell adhesion molecule
PFA	Paraformaldehyde
ROSA	Reverse orientation splice acceptor
SCID	Severe combined immunodeficient
