# Supplementary material for: Assessing the Effectiveness of a Far-Red Fluorescent Reporter for Tracking Stem Cells In Vivo
Source: Int J Mol Sci. 2017 Dec 22;19(1):19. doi: 10.3390/ijms19010019 (PMC5795970; doi:10.3390/ijms19010019)
Supplement: Supplementary file 1 [file ijms-19-00019-s001.pdf]

Supplementary Materials: 'Assessing the Effectiveness of a Far-red Fluorescent Reporter for Tracking Stem Cells *In Vivo*' by Zhou *et al*

Table S1. Tumour monitoring record post injection of *Bra-GFP/Rosa26-E2C* mESCs

**Mouse No.**	**Cell Lines**	**Cell No.** (Million)	**Day 4**				**Day 7**				**Day 9**			
			**Wt** (g)	**L** (mm)	**W** (mm)	**H** (mm)	**Wt** (g)	**L** (mm)	**W** (mm)	**H** (mm)	**Wt** (g)	**L** (mm)	**W** (mm)	**H** (mm)
1	E2C	10	19.34	5.1	6.2	2.0	19.15	N/A	N/A	N/A	19.52	N/A	N/A	N/A
		7.5		N/A	N/A	N/A		N/A	N/A	N/A		6.4	4.5	3.5
		5		N/A	N/A	N/A		N/A	N/A	N/A		4.4	4.4	2.2
	Ctrl	10		N/A	N/A	N/A		6.8	6.7	4.0		9.2	9.0	7.7
2	E2C	10	17.00	3.4	4.4	2.0	17.55	3.6	3.5	3.0	17.72	5.6	4.7	3.2
		7.5		N/A	N/A	N/A		5.2	5.3	3.0		9.6	8.9	6.4
		5		N/A	N/A	N/A		4.0	4.9	3.0		9.0	6.8	4.7
	Ctrl	10		N/A	N/A	N/A		3.6	3.3	3.0		5.4	5.4	3.0
3	E2C	10	18.20	5.8	6.0	2.0	20.45	8.5	5.2	3.0	20.88	9.9	9.7	5.3
		7.5		N/A	N/A	N/A		7.0	7.0	3.0		9.7	9.6	6.7
		5		N/A	N/A	N/A		3.0	3.0	1.0		4.8	3.4	1.0
	Ctrl	10		N/A	N/A	N/A		3.0	3.0	1.0		5.5	4.8	3.2

(Wt, weight; L, length; W, width; H, height; E2C, *Bra-GFP/Rosa26-E2C* mESCs; Ctrl, untransfected *E14-Bra-GFP* mESCs)

Table S2. Tumour volume and *in vivo* monitoring record post injection of *Bra-GFP/Rosa26-E2C* mESCs

Cell Lines	Cell No. (Million)	Mouse No.	**ROI Radiance (e+08)** (p/sec/cm²/sr)					**Volume** (mm³)		
			**Day 0**	**Day 1**	**Day 4**	**Day 7**	**Day 9**	**Day 4**	**Day 7**	**Day 9**
E2C	10	1	2.127	1.368	2.376	1.881	1.834	33.11	0.00	0.00
		2	2.142	1.305	2.275	2.276	2.480	15.67	19.79	44.10
		3	2.723	1.908	4.135	3.933	3.122	36.44	69.43	266.49
		Mean	2.331	1.527	2.929	2.697	2.479	28.41	29.74	103.53
			±0.340	±0.331	±1.046	±1.089	±0.644	±11.159	±35.768	±142.840
	7.5	1	2.657	1.780	2.981	3.129	3.109	0.00	0.00	52.78
		2	2.243	1.447	2.983	2.639	2.749	0.00	43.29	286.31
		3	1.742	1.174	2.330	2.860	2.416	0.00	76.97	326.68
		Mean	2.214	1.467	2.813	2.876	2.758	0.00	40.09	221.92
			±0.458	±0.303	±0.376	±0.245	±0.347	±0.000	±38.584	±147.866
	5	1	2.180	1.580	2.459	2.528	2.546	0.00	0.00	22.30
		2	1.821	1.318	2.869	2.017	2.127	0.00	30.79	150.61
		3	1.705	1.659	2.475	2.429	2.608	0.00	4.71	8.55
		Mean	1.902	1.519	2.601	2.325	2.427	0.00	11.83	60.48
			±0.248	±0.178	±0.232	±0.271	±0.262	±0.000	±16.583	±78.351
Ctrl	10	1	1.419	1.115	1.849	1.459	1.475	0.00	95.42	333.83
		2	1.826	1.448	2.933	1.895	2.227	0.00	18.66	45.80
		3	2.122	1.357	3.517	2.361	2.861	0.00	4.71	44.23
		Mean	1.789	1.307	2.766	1.905	2.188	0.00	39.60	141.29
			±0.353	±0.172	±0.846	±0.451	±0.694	±0.000	±48.844	±166.744

(ROI, region of interest; E2C, *Bra-GFP/Rosa26-E2C* mESCs; Ctrl, untransfected *E14-Bra-GFP* mESCs)
